# The Pandora's box of predatory journals

**DOI:** 10.17179/excli2022-6011

**Published:** 2023-03-24

**Authors:** Lucindo J. Quintans-Júnior, Adriano A.S. Araújo, Robério R. Silva, Paulo R. Martins-Filho

**Affiliations:** 1 Graduate Program of Health Science (PPGCS), Federal University of Sergipe (UFS). Aracaju, Sergipe, Zip code: 49100-000, Brazil; 2 Graduate Program in Animal Science (PPZ), State University of Southwest Bahia (UESB). Itapetinga, Bahia, Zip code: 45.700-000, Brazil

## ⁯⁯⁯⁯

At a time when the debate on scientific integrity is gaining more space, the issue of predatory journals returns to the stage as one of the main actors. The discussion about predatory journals has become more prevalent in the scientific community due to the growing number of suspect scientific journals and the lack of a clear definition to define them (Grudniewicz et al., 2019[3]). Despite the lack of consensus how to define them, the predatory journal can be well-marked as a publication that claims to be a legitimate academic journal, but engages in unethical and unprofessional practices solely for the purpose of making a profit, often without any consideration for the quality of the research published (Elmore and Weston, 2020[2]).

Thus, predatory journals typically have low or nonexistent academic standards for peer review, often accepting papers with little to no scrutiny. They may also charge exorbitant fees to authors, without providing the support or resources necessary to ensure high-quality publications (Grudniewicz et al., 2019[3]). These journals may also engage in aggressive marketing tactics, spamming researchers with unsolicited emails and invitations to submit their work. Since new predatory journals can emerge at any time and the definition of what constitutes a predatory journal is subjective, it is a challenge to determine their exact number. However, several studies have attempted to estimate their prevalence, with a study published in the journal BMC Medicine (in 2015) estimating that there were over 11,000 such journals (Shen and Björk, 2015[4]).

There is a widely accepted consensus that predatory journals are deleterious to the development of ethical science based on scientific integrity. However, it is sometimes difficult to separate their egregious practices from those of, what is, in effect, a lucrative casino that the big publishers operate. Many of these publishers, such as Elsevier, Wiley, and Springer Nature have profit margins that exceed those of major tech companies like Apple and Google, with some publishers having profit margins of 30-40 % or higher (Buranyi, 2017[1]).

A significant number of big publishers charge fees that can reach thousands of dollars to publish an article in the Open Access system, which is an indisputably lucrative market for them. This system is anachronistic, unhealthy, and counterproductive for researchers who must pay to have their work published in journals deemed "more respectable" because they belong to large publishers. While many of these journals were originally established by respected scientific societies and feature peer-reviewed papers, they have become a 'big business' that primarily benefits the publishers instead of the researchers. What is even more outrageous is that researchers themselves work for free - considering it part of their duty to the academic community - as reviewers for these rich journals, while having to pay to have their papers published.

Moreover, funding agencies, who pay for the research on which studies and articles are based, have to provide the funds not only for researchers to submit articles but also to pay for access to articles that support their work, becoming an unsustainable and irrational process from the researcher's point of view; Meanwhile the big publishers rack up profits made from the science developed in their countries. This practice is neither reasonable nor rational, yet the big publishers have the power to stifle the debate on the subject. How many articles can you find on the subject in leading journals produced by big publishers? You could probably count the number of good articles on the fingers of one hand.

In respect of predatory journals, one way out to reduce their number would be for scientific societies to set up working groups and, using their expertise, better direct the researchers in their field, helping them to identify and avoid these types of journals. In respect of the big publishers, dialogue between the main actors involved in the publication process is urgently required to expand the reach and access to published science. What has become the "paper publishing market" is an untenable system that works against researchers, impoverishes science, and undermines the scientific ecosystem, thereby making society vulnerable by acting as a brake on scientific advancement.

Therefore, it is imperative to open this Pandora's box and have researchers, funding agencies, scientific societies, and publishers sit at the table to find a more sustainable solution that prioritizes scientific integrity, ethical principles, and the security of scientific information. In a world faced by a growing number of challenges, we need a system that produces reliable data and encourages quality research, not a “big casino” run by wealthy players acting as the gatekeepers in respect of the publication of the results of scientific studies.

## Conflict of interest

The authors declare no conflict of interest.
